# Percutaneous cerclage wiring for the surgical treatment of displaced patella fractures

**DOI:** 10.1007/s11751-014-0184-0

**Published:** 2014-02-05

**Authors:** Tomoji Matsuo, Taiji Watari, Kiyohito Naito, Atsuhiko Mogami, Kazuo Kaneko, Osamu Obayashi

**Affiliations:** 1 Department of Orthopaedic Surgery, Juntendo University Shizuoka Hospital, 1129, Nagaoka, Izunokuni, Shizuoka 410-2295 Japan; 2 Department of Orthopaedic, Juntendo University, 2-1-1, Hongo, Bunkyo-ku, Tokyo 113-8431 Japan

**Keywords:** Percutaneous cerclage wiring, Patellar fractures, Soft tissue around patella, Post-operative displacement

## Abstract

The patella plays an important role in the knee joint extension, and a patella fracture requires surgical treatment when it is accompanied by displacement of bone fragments and a joint surface gap. In patella fractures, there is disruption of the soft tissue structures that support the knee extension mechanism. We use a method of percutaneous cerclage wiring to fix the patella and include the peripatellar soft tissues in five patients. All cases were closed fractures, and the AO classification was type A in 1 and type C in 4. At a mean follow-up of 11.2 months, union was achieved in four cases with failure in one inferior pole fracture avulsion. There was no extensor lag noted in any patient, with mean flexion at 141° (120–160). As this percutaneous cerclage wiring method includes soft tissue approximation in the wiring, it may be especially suitable for comminuted fractures for which classic tension band wiring techniques cannot be used. We employed this procedure to atraumatically manipulate peripatellar soft tissues together with the fracture fragments in order to obtain optimal restoration of continuity of the extensor mechanism.

## Introduction

The patella is the largest sesamoid bone in the human body and has a major role in extension of the knee. Patella fractures occur through excessive tension or by a direct external force, both disrupting knee extension. Surgical treatment is necessary for displaced fractures to restore extension and reduce the risk of potential patellofemoral (PF) osteoarthritis (OA) [1–5].

Tension band wiring and screw fixation are osteosynthesis methods for patella fractures. However, these surgical procedures are difficult if the fracture is highly comminuted [6]. The roles of peripatellar soft tissues in patella stability suggest that, in addition to reduction and fixation of the patella fracture, repair of the peripatellar soft tissues is important [1, 6–9]. In accordance with this principle, we have used a percutaneous cerclage wiring method to include repair of the peripatellar soft tissues. We report the clinical outcomes and pitfalls of this surgical procedure in 5 patients.

## Patients and methods

There were two male and three female patients; the mean age was 50.4 years (24–73 years). All underwent surgery at our hospital and were followed-up for 4 months or longer. The study was approved by the local ethical committee (Juntendo University Shizuoka Hospital, Shizuoka, Japan) and was conducted in accordance with the Declaration of Helsinki and Ethical Guidelines for Epidemiological Research. All patients provided written informed consent for the participation in the study. The cause of injury was a stumble in 3, fall in 1 and a traffic accident in 1. All cases were caused by a direct external force and were closed fractures; the AO classification was type A in 1 and type C in 4. The fracture pattern was a simple transverse fracture in 3- and a 4-part comminuted fracture in 2. All patients underwent osteosynthesis employing percutaneous cerclage wiring within 8 (range 2–8) days of injury (Table 1).

**Table 1:** Our series of five patients operated by percutaneous cerclage wiring

	Sex	Age	Side	Direct or indirect	Open or close	AO classification	Type	Delay (days)	Past history
1	F	38	Lt	Direct	Close	C	2 part	8	–
2	F	64	Rt	Direct	Close	C	4 part	5	DM Depression HT
3	F	53	Rt	Direct	Close	C	2 part	5	–
4	M	24	Rt	Direct	Close	C	4 part	8	–
5	M	73	Lt	Direct	Close	A	2 part	2	HCC DM

*DM* diabetes mellitus; *HT* hypertension; *HCC* hepatocellular carcinoma

**Fig. 1:**
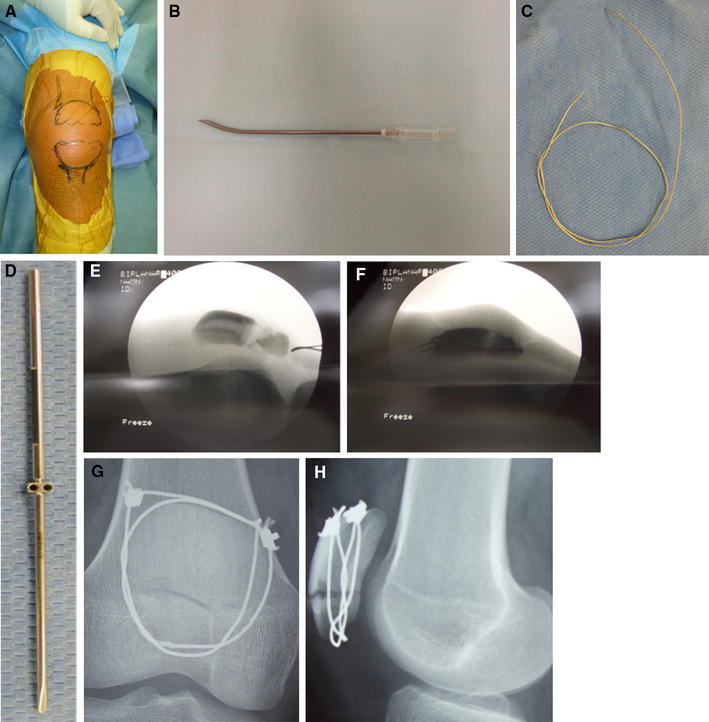
Surgical technique. **a** Ten-millimetre and 5-mm small medio-lateral skin incisions at the superior and inferior margins of the patella, respectively. **b** The tip of the inner tube of a Sur-flo (HAKKO ELASTER TYPE-1, HAKKO Co., Ltd., Tokyo, Japan) was bent and used as a cable passer. **c** Cable, **d** sleeve: for these, the AI-wiring system (AI-medic Co., Ltd., Tokyo, Japan) was used. **e** The cable passer was inserted into the patellar tendon through the skin incision at the inferior margin of the patella. **f** The cable was applied around the patella including the soft tissues, penetrating through the quadriceps femoris tendon on the proximal side, and then passed along the superior margin of the patella. **g** Frontal view after fixation on plain X-ray radiography, **h** lateral view after fixation on plain X-ray radiography: two cable wires were passed through the shallow and deep layers, respectively

Surgery was performed under spinal anaesthesia and in the supine position with slight flexion of the knee joint. Ten-millimetre and 5-mm medio-lateral skin incisions were made at the superior and inferior margins of the patella, respectively (Fig. 1a). The tip of the inner tube of a 14G Sur-flo (Hakko Elaster Type-1, Hakko Co., Ltd., Tokyo, Japan) cannula was bent and used as a cable passer (Fig. 1b). For the cable, the AI-wiring system (AI-medic Co., Ltd., Tokyo, Japan) was used (Fig. 1c, d). Firstly, the cable passer was inserted into the patellar tendon through the skin incision at the inferior margin, followed by the passage of the cable (Fig. 1e). The cable was passed around the patella including the soft tissues, penetrating through the quadriceps femoris tendon proximally, and then passed along the superior margin of the patella (Fig. 1f). This cable wiring was applied in each of the superficial and deep layers (Fig. 1g, h). On realignment of the patellofemoral joint, congruency of the patella to the knee joint was confirmed by bending and extending it. Tension was applied using a cable tensioner to lock the sleeve. Post-operatively, range-of-motion training of the knee joint was initiated on the day following surgery and limited by the patient’s comfort. In addition, full weight-bearing with the knee joint held in extension was permitted.

The range of motion of the knee joint at the final follow-up, time required to bone union on plain X-ray radiography, X-ray features of osteoarthritis of the patellofemoral joint and complications were recorded.

## Results

The mean duration of follow-up was 11.2 months (4–21 months). No limitation of extension was noted in any patient with average flexion at 141° (120–160). Excluding the AO type A case, which was an inferior pole fracture, bone union was achieved in the other four cases. No osteoarthritis of the PF occurred throughout the course.

One superficial infection occurred and was treated with oral antibiotics. On bone union, inflammation resolved by removing the implants. Implant fracture occurred in two patients. Since it occurred after bone union in one case, the implants were removed. For the other case, which was the AO type A inferior pole fracture, a re-displacement occurred after implant fracture. As this patient had an underlying diagnosis of hepatocellular carcinoma, revision surgery was not performed and treatment continued without bone union (Table 2).

**Table 2:** Results of our series of five patients operated by percutaneous cerclage wiring

	Delay of F/U (month)	ROM	Bone healing	PF OA	Complication
1	11	0-0-140	Union	–	–
2	8	0-0-120	Union	–	–
3	12	0-0-135	Union	–	Breakage of implant
4	21	0-0-150	Union	–	Superficial infection
5	4	0-0-160	Non-union	–	Breakage of implant Fixation failure

*F/U* follow-up; *ROM* range of motion; *PF* patellofemoral joint; *OA* osteoarthritis

## Discussion

In addition to the quadriceps femoris tendon and femoral fascia attached to the superior pole of the patella and the patellar tendon at the inferior pole, the femoral fascia, vastus medialis muscle and vastus lateralis tendon form the patellar retinaculum to aid extension of the knee. The patella works as a lever arm by separating the point of action of the knee extension system from the rotation centre to increase the extension efficiency [1]. The patella is moved like a pulley in the femoral sulcus by the actions of the quadriceps femoris and patellar tendon with the bony support augmented by medio-lateral soft tissue restraints [6, 7]; the medial patellofemoral ligament (MPFL), medial patellomeniscal ligament (MPML) and medial patellotibial ligament (MPTL) are closely involved in the medial support system, with the MPFL having the most important role [8]. The lateral support system is complex and divided into two layers: a superficial layer comprising of fibrous tissue extending from the anterior vastus lateralis muscle and posterior oblique retinaculum, and a deep layer comprising the patellar epicondylar ligament, deep transverse retinaculum and patellotibial ligament directly attached to the inferior pole of the patella. In addition, the iliotibial ligament is also involved in the stability of lateral support [9]. Therefore, in addition to the quadriceps femoris and patellar tendons involved in extension and flexion of the knee joint, many support systems involving soft tissues are circumferentially present around the patella.

Percutaneous cerclage wiring for patella fracture can be applied through several small skin incisions without dissecting the fractured region and surrounding soft tissues and provides a minimally invasive approach to this procedure. In addition, range-of-motion training can be started early after surgery because the soft tissues are included in wire fixation. All five patients started continuous passive motion (CPM) on the day following surgery. Furthermore, favourable bone union was achieved even in the 2 comminuted fracture cases, suggesting that this procedure is useful for comminuted fractures for which classic tension band wiring is not applicable (Cases 2 and 4, Table 1). In comminuted fracture cases, an early start of range-of-motion training may facilitate indirect reduction through 3-point bending forces loaded on the patellofemoral joint in flexion and extension of the knee joint, even if the patellofemoral joint surface cannot be accurately reduced [1, 6]. Bone union was achieved in 4 of the 5 cases with no osteoarthritis of the patellofemoral joint seen. We suggest that percutaneous osteosynthesis which includes stabilization of the soft tissues but performed without extensive dissection is more appropriate for severely comminuted fractures.

Another advantage of not dissecting the soft tissues is early acquisition of a favourable range of motion after surgery. Wu et al. [10] applied tension band wiring and had a measured knee joint flexion angle of 138.9° (110–140) after surgery. Chang et al. [11] applied tension band wiring through cannulated screws and had a knee joint flexion angle of 123° (100–140). Our patients, treated with percutaneous cerclage wiring, had a knee joint flexion angle of 141° (120–160), suggesting that a similar range of motion to those reported was achieved without formal open reduction in the patellofemoral joint surface. Complications of open reduction and internal fixation (ORIF) include knee joint contracture particularly in extension as adhesions between the quadriceps femoris muscle and femur at the proximal patella are implicated [12, 13]. Since no dissection is applied to the soft tissues in this procedure, adhesions causing post-operative contracture are unlikely to occur.

For simple 2-part patella fractures, it is possible that osteosynthesis with screws or classic tension band wiring obtains better fixation, but in cases of comminuted fractures, this method can be difficult. For these reasons, the method of percutaneous cerclage wiring, including the soft tissues around fractured patella, is an effective alternative. Moreover, even if some minor irregularity of the articular surface of the patella remains, the early commencement of range-of-motion exercises after surgery may cater for a ‘remoulding’ within the femoral groove.

**Fig. 2:**
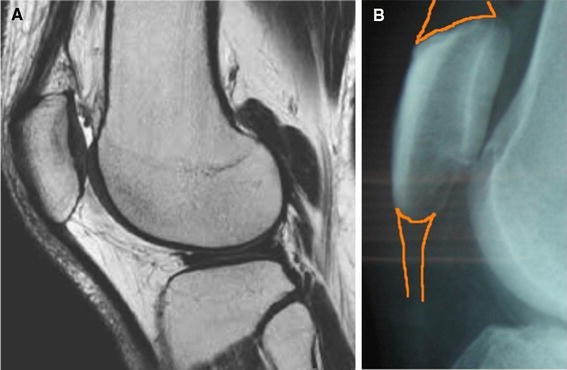
Relationship between the patella and quadriceps femoris and patellar tendons. **a** MRI, **b** fluoroscopy: the quadriceps femoris tendon is attached anteroposteriorly to the patella widely at the superior margin but narrow at the inferior margin

**Fig. 3:**
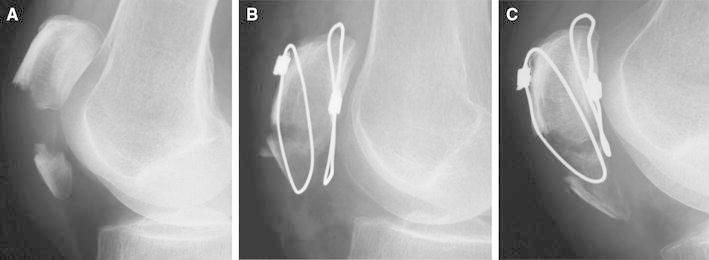
Displaced case (Case 5). **a** Preoperative plain X-ray radiography: AO type A, 2-part fracture. **b** The patellar tendon was not caught by the wire in the deep layer, and the fractured region was supported only by the wire in the shallow layer. **c** Plain X-ray radiography 4 months after surgery: the fractured region was displaced

Post-operative displacement occurred in one patient. The quadriceps femoris tendon is attached to the patella across a wide surface at the superior margin, but, in contrast, the patella tendon is attached across a narrower width at the inferior margin (Fig. 2a, b). In the AO type A case (Case 5) in which displacement occurred after surgery (Table 1; Fig. 3a), the patellar tendon was not captured by the wire in the deep layer on review of the post-operative plain X-ray and, as such, the fractured region was supported only by the wire in the shallow layer (Fig. 3b), resulting in displacement of the fracture 4 months after surgery (Fig. 3c). To prevent complications caused by this procedure, we have redesigned the skin incision at the inferior margin of the patella as near the patellar tendon as possible, while imaging the patellar tendon laterally under fluoroscopy (Fig. 2b) to allow insertion of the passer under the inferior pole of the patella.

## Conclusion

We performed osteosynthesis employing percutaneous cerclage wiring for patella fractures in five patients. Displacement of the fractured region occurred after surgery in one patient, but bone union was achieved in the other four patients and a favourable range of motion of the knee joint was acquired. We employed this procedure to atraumatically manipulate the peripatellar soft tissues. Although this procedure is not simple, it may be superior to classic fixation techniques for highly comminuted fractures with minimal displacement.
